# A novel method of measuring leaf epidermis and mesophyll stiffness shows the ubiquitous nature of the sandwich structure of leaf laminas in broad-leaved angiosperm species

**DOI:** 10.1093/jxb/erv024

**Published:** 2015-02-11

**Authors:** Yusuke Onoda, Feike Schieving, Niels P. R. Anten

**Affiliations:** ^1^Section of Plant Ecology and Biodiversity, Institute of Environmental Sciences, Utrecht University, P.O. Box 800.84, 3508TB Utrecht, The Netherlands; ^2^Department of Agriculture, Kyoto University, Kyoto 606-8502, Japan; ^3^Centre for Crop Systems Analysis, Wageningen University, P.O. Box 430, 6700AK Wageningen, The Netherlands

**Keywords:** Biodiversity, biomechanics, cuticle, epidermis, evolution, leaf anatomy, mechanical design, mesophyll, sandwich structure, turgor pressure.

## Abstract

Plant leaves commonly exhibit a thin, flat structure that facilitates a high light interception per unit mass, but may increase risks of mechanical failure when subjected to gravity, wind and herbivory as well as other stresses. Leaf laminas are composed of thin epidermis layers and thicker intervening mesophyll layers, which resemble a composite material, i.e. sandwich structure, used in engineering constructions (e.g. airplane wings) where high bending stiffness with minimum weight is important. Yet, to what extent leaf laminas are mechanically designed and behave as a sandwich structure remains unclear. To resolve this issue, we developed and applied a novel method to estimate stiffness of epidermis- and mesophyll layers without separating the layers. Across a phylogenetically diverse range of 36 angiosperm species, the estimated Young’s moduli (a measure of stiffness) of mesophyll layers were much lower than those of the epidermis layers, indicating that leaf laminas behaved similarly to efficient sandwich structures. The stiffness of epidermis layers was higher in evergreen species than in deciduous species, and strongly associated with cuticle thickness. The ubiquitous nature of sandwich structures in leaves across studied species suggests that the sandwich structure has evolutionary advantages as it enables leaves to be simultaneously thin and flat, efficiently capturing light and maintaining mechanical stability under various stresses.

## Highlight

Leaf laminas are designed in sandwich structure, a very efficient structure used in e.g. airplane wing, which enables leaves to be thin, flat, ideal for photosynthesis but also mechanically stable.

## Introduction

The primary function of plant leaves is recognized as photosynthesis and has been studied intensively from various points of view (Lambers *et al.*, 2008; Blankenship, 2014). However, it is much less recognised that a large fraction (i.e. 14–77%) of leaf dry mass is in structural components i.e. cell walls (Onoda *et al.*, 2011). The large investment in structural mass is considered to physically protect the photosynthetic leaf function from a suite of stressors including gravity, wind and herbivory (Read and Stokes 2006). Elucidating how the leaf anatomical structure is built in relation to these basic physical requirements is essential to better understand the functioning of leaves.

Leaves typically have a flat, thin structure, which is associated with a large leaf surface area per unit biomass, and is thus ideal for efficient light interception (Givnish, 1986; Braybrook and Kuhlemeier, 2010), but are concomitantly prone to mechanical failure due to their thin structure (Niklas, 1999; Read and Stokes, 2006). Therefore leaves should be designed in a way that they are not only thin but also reasonably stiff and strong. In engineering, such demands are typically met by designing objects as sandwich structures.

A typical sandwich structure is composed of stiff outer surfaces and a lightweight core, which greatly increases specific stiffness (stiffness per unit mass) in bending and has been used in many engineering constructions such as airplane wings and surfboards (Gibson *et al.*, 1988; Gere and Timoschenko, 1999). Leaf laminas resemble a sandwich structure in that they are composed of two layers of epidermis tissue and intervening mesophyll tissues (Vincent, 1982; Gibson *et al.*, 1988; Niklas, 1991, 1992, 1999; Moulia *et al.*, 1994; Moulia and Fournier, 1997). This design may be an evolutionary solution to increase lamina robustness with minimum biomass investment. It has been shown that the lamina (as well as veins) plays a significant role in whole leaf mechanical stability. For instance, Moulia *et al.* (1994) reported in maize that the lamina itself contributed about 50% to leaf bending stiffness at the middle of leaves and more towards the leaf tips. Similar importance of lamina stiffness in leaf bending stiffness was shown by a finite element method (Kobayashi *et al.*, 2000, 2002). In addition to the importance of leaf laminas in maintaining whole leaf plane structure, a sandwich structure has also advantages in protecting leaves from herbivory and pathogen attack by its stiff and strong surface (Grubb, 1986).

Even though the sandwich structure of leaf laminas has been alluded to in several studies and its potential advantage for plants is recognized (Vincent, 1982; Gibson *et al.*, 1988; Niklas 1991, 1992, 1999; Moulia *et al.*, 1994; Moulia and Fournier, 1997; Niinemets and Fleck, 2002), to our knowledge no study has actually determined to what extent leaf laminas are indeed designed as a sandwich structure. It is therefore also unknown how common this design is among different plant species. Such an assessment requires knowledge of the mechanical properties of the epidermis and mesophyll layers. However, we know of only one study that evaluated the mechanical properties of the epidermis and mesophyll layers (Gibson *et al.*, 1988). They indirectly estimated the Young’s modulus (intrinsic material stiffness) of each layer by assuming stiffness of the epidermis and mesophyll layers to be proportional to their respective cell wall cross-sectional areas in *Iris* leaves. The lack of knowledge for stiffness of leaf epidermis and mesophyll layers may be due to the practical difficulty in isolating leaf epidermis layers from other tissue. Furthermore, separating tissues from a turgid leaf may release tissue stress (also called residual stress in engineering), and could have confounding effects on the estimate of Young’s modulus of tissues (Hejnowicz and Sievers, 1995*a*).

In this study, we first quantified the extent to which leaf laminas behave as sandwich structures. To do so, we developed a novel method to quantify the Young’s moduli of the epidermis and mesophyll layers without separating these layers, based on linear elastic theory (Gere and Timoschenko, 1999). As discussed later, biological materials are much more complicated than an ideal material that follows linear elastic theory (Baskin and Jensen, 2013), yet we believe that this approach is an important first step to evaluate the mechanical properties of the epidermis and mesophyll layers. By applying this method to a diverse set of 36 species, we determined to what extent sandwich structures are common across species (i.e. herbaceous, woody deciduous or woody evergreen) and across phylogeny. Furthermore, the variation in the Young’s moduli of the epidermis and mesophyll layers was analysed with respect to leaf morphology (e.g thickness and tissue density) and anatomy (e.g. mesophyll, epidermis, cuticle and cell walls) to better understand how leaf structures are functionally built in relation to the multiple requirements for photosynthesis, mechanical stability and defence.

## Materials and methods

### Model

In the present study, we use a few material terms that may not be commonly used in plant science, so first we explain these terms to avoid possible confusions. Young’s modulus is an intrinsic mechanical property of the material independent of its geometry (see equation 9 for the calculation). Tensile stiffness and bending stiffness are structural properties of a material, which depend on the Young’s modulus and geometry of the material (see also equations 1 and 2, Gere and Timoschenko 1999).

Many leaves have a flat plane structure consisting of two layers of epidermis and an intervening mesophyll core, which could be approximated by a sandwich structure as described below. Some other leaves have more complicated structures such as cylinder or shell structure, which require a more complicated mechanical modelling and are not considered in this study.

**Fig. 1.**
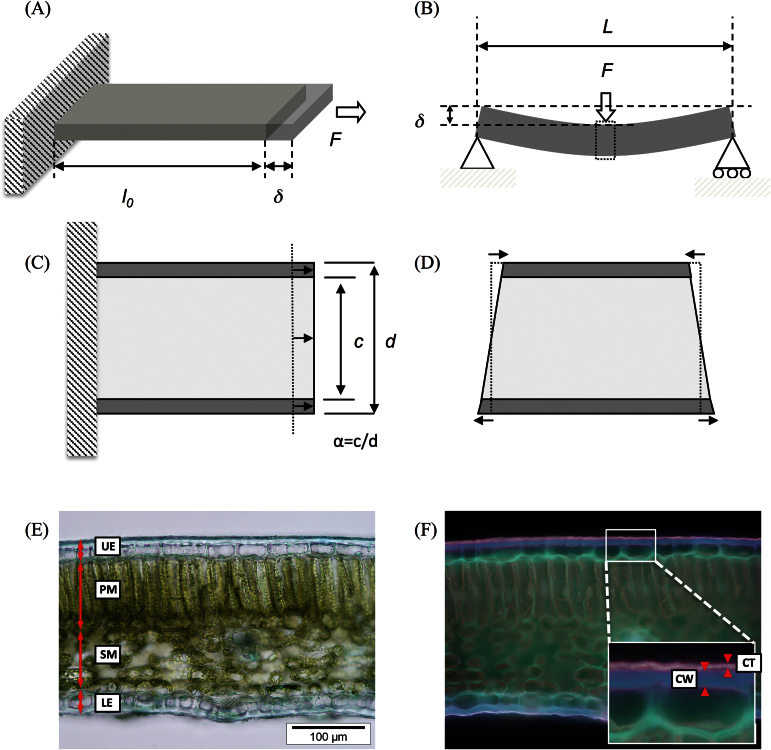
A schematic drawing of the tensile test (A) and bending test (B), and how a sandwich structure material is deformed under tension (C) and bending (D) from the side view. The part shown in the dotted box in (B) is enlarged in (D). Representative images of leaf lamina cross-sections (*Helleborus orientalis*) with transmitted light (E) and UV fluorescence (F). Cell walls (CW) and cuticles (CT) are more visible in the UV fluorescence image. Abbreviations: *F*, force applied to the specimen; *l*_0_, original length of the specimen; *δ* displacement; *L*, span length; *c*, thickness of the core; *d*, thickness of the whole sandwich structure; UE, upper epidermis layer; PM, palisade mesophyll layer; SM, spongy mesophyll layer; LE, lower epidermis layer. (This figure is available in colour at *JXB* online.)

For a simple sandwich structure, such as a plane leaf lamina, longitudinal tensile stiffness of the sandwich structure in the tensile test can be expressed as a sum of the longitudinal Young’s moduli of the face (*E*_f_) and core (*E*_c_) weighted by cross-section area of each layer, according to the superposition principle under the linear elastic theory (Gere and Timoschenko, 1999) (Fig. 1).

$$E_{T}A=E_{f}A_{f}+E_{c}A_{c}$$ (1)

where *A* is cross-section area of the composite sandwich structure, and *A*_f_ and *A*_c_ are respectively the cross-section area of the face (i.e. epidermis) and core (i.e. mesophyll) layers (*A*=*A*_f_+*A*_c_).

Similarly, bending stiffness of the sandwich structure can be expressed as a sum of *E*_f_ and *E*_c_ weighted by the second moment of area of each layer (Gere and Timoschenko, 1999). The second moment of area is a geometrical property of a beam and depends on cross-section area and shape (described later).

$$E_{B}I=E_{f}I_{f}+E_{c}I_{c}$$ (2)

where *I* is the second moment area of the composite sandwich structure, and *I*_f_ and *I*_c_ are the second moment of area of the epidermis and mesophyll layers relative to the neutral axis of the leaf (*I*=*I*_f_+*I*_c_).

*A*_c_/*A* is equal to relative thickness of the mesophyll in the whole lamina thickness, α (0<α<1, Fig. 1) and *A*_f_/*A* is thus equal to 1-*α*. From these, equation 1 can be rewritten as follows:

$$E_{T}=\left(1-\alpha \right)E_{f}+\alpha E_{c}$$ (3)

For the bending test, the second moment of area of a layer (full and symmetric relative to the neutral axis) is proportional to the third power of its thickness (Gere and Timoschenko, 1999). Therefore the second moment of area of the mesophyll layer (*I*_c_) is equal to *α* ^3^ *I*, and by the principle of superposition, the second moment of area of the epidermis layer (*I*_f_) is equal to (1-*α* ^3^) *I.* Therefore equation 2 can be expressed as follows:

$$E_{B}=\left(1-\alpha^{3}\right)E_{f}+\alpha^{3}E_{c}$$ (4)

From equations 3 and 4, *E*_B_/*E*_T_ can be determined as follows:

$$\frac{E_{B}}{E_{T}}=\frac{1-\alpha^{3}(1-\beta )}{1-\alpha (1-\beta )}$$ (5)

where *β* is *E*_c_/*E*_f_.

**Fig. 2.**
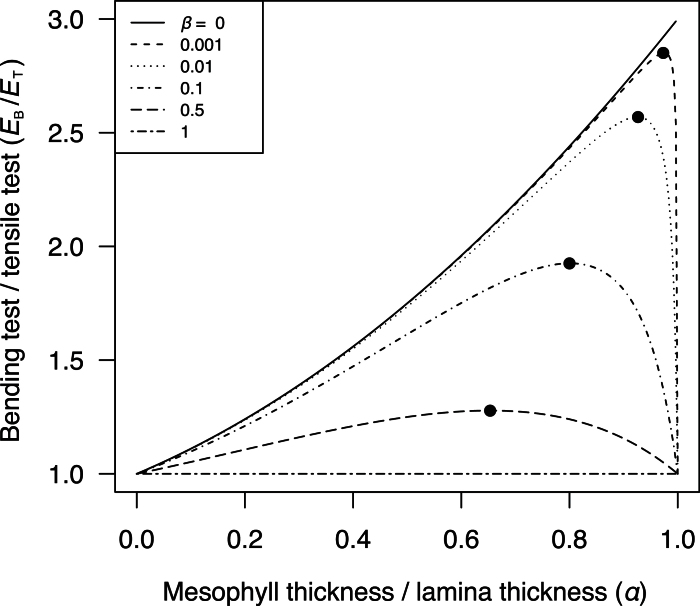
Theoretical relationships between *E*_B_/*E*_T_ (the ratio of Young’s moduli measured by bending test to that measured by tensile test) and *α* (the fraction of mesophyll layer in leaf lamina) at variable *β* (the ratio of the mesophyll layer Young’s modulus to the epidermis layer Young’s modulus) as calculated from $\frac{E_{B}}{E_{T}}=\frac{1-\alpha^{\text{3}}\text{(1}-\beta \text{)}}{1-\alpha \text{(1}-\beta \text{)}}$ Filled circles denote the optimal *α* that maximizes *E*_B_/*E*_T_ at a given *β*. Note that when the epidermis layer is extremely thin and stiff compared to the mesophyll layer (α≈1, β≈0), *E*_B_/*E*_T_ approaches to the value of 3.

The relationship between *E*_B_/*E*_T_, *α* and *β* is shown in Fig. 2. If a material is homogeneous (*β*=1), *E*_B_/*E*_T_ is equal to 1. This was validated through measurements on filter paper samples (No. 595, Whatman, Maidstone, UK), a homogenous material, showing *E*_B_/*E*_T_ = 0.996±0.17 (mean ± SD, *n*=12). By contrast, if the face layers are stiffer than the core (*β*<1) as would be expected for an efficient sandwich structure, *E*_B_/*E*_T_ is higher than 1 (Fig. 2). Note that the optimal α that maximises *E*_B_/*E*_T_ increases with decreasing *β* (Fig. 2). In the extreme case, where the face layers are exclusively stiffer than the core (*β*≈0), equation 5 can be expressed as follows:

$$\frac{E_{B}}{E_{T}}\approx 1+\alpha +\alpha^{2}$$ (6)

This equation shows that the theoretical maximum of *E*_B_/*E*_T_ for an ideal sandwich structure with an extremely thin and stiff face layers (*α*≈1, *β*≈0), the *E*_B_/*E*_T_ ratio can increase up to 3.

In equations 3 and 4, *E*_c_ and *E*_f_ cannot be directly measured, however because there are two equations and only two unknowns, these two variables can be solved analytically (see SI for the full derivation).

$$E_{f}=\frac{E_{B}-\alpha^{2}E_{T}}{1-\alpha^{2}}$$ (7)

$$E_{c}=\frac{\left(1+\alpha +\alpha^{2}\right)E_{T}-\;E_{B}}{\alpha \left(1+\alpha \right)}$$ (8)

Equations 7 and 8 show that Young’s moduli of the epidermis layers (*E*_f_) and that of the mesophyll layer (*E*_c_) can be estimated from only three measurable variables: (i) Young’s modulus of the lamina measured by tensile tests (*E*_T_), (ii) Young’s modulus of the lamina measured by bending tests (*E*_B_) and (iii) the fraction of lamina cross-section composed of the mesophyll layer (*α*), which can be determined microscopically (see Methods section ‘Anatomy analysis’).

One may argue that when the bulk core material is too compliant, there is a possibility of buckling and subsequent delamination of the faces upon bending. This effect may be negligible in leaves since the mesophyll tissues are turgid and the bundle sheath extensions, which resemble a honeycomb structure, keep a distance between the two epidermis layers.

### Plant materials

We collected leaves from 36 broad-leaved species (33 different families) including 15 herbaceous species, 12 deciduous woody species and 9 evergreen woody species (Table 1) that were grown outdoors in the Utrecht University Botanical Garden (latitude=52.087N, longitude=5.167W) on 13 September 2006. All sampled leaves were fully developed without any visual damage or senescence. These species were collected to cover a wide range of angiosperm species including magnoliids, monocots and a wide variety of eudicots (Supplementary Fig. S1). However very small, highly dissected or twisted leaves were avoided as these leaves could not be used for the mechanical measurements. Each sampled leaf was wrapped in a wet paper towel, and five or more leaves per species were combined and sealed in a plastic bag, and stored at 4°C to avoid loss of turgor pressure until just before the mechanical measurements.

**Table 1.** Species information, leaf mechanical properties and morphology of 36 angiosperm species Abbreviations are as follows; FG, functional group; H, herbaceous species; W, woody species; D, deciduous; E, evergreen; E_T_, Young’s modulus measured by the tensile test (MPa); E_B_, Young’s modulus measured by the bending test (MPa); Th, leaf thickness (mm); *α,* mesophyll thickness per unit lamina thickness (m m^-1^); E_f_, Young’s modulus of epidermis layer (MPa); E_c_, Young’s modulus of mesophyll layer (MPa); E_B_I, bending stiffness per unit lamina width (N mm^3^). Means of five replications are shown.

Species	Family	FG	*E*_T_	*E*_B_	*E*_B_/*E*_T_	Th	*α*	*E*_f_	*E*_c_	*E*_B_ *I*
*Amaranthus hybridus*	Amaranthaceae	H/D	8.3	23.6	2.91	0.229	0.792	50	-2.6	0.023
*Arabidopsis thaliana*	Brassicaceae	H/D	2.4	4.9	2.04	0.206	0.802	11	0.8	0.004
*Bergenia* ‘Abendglut’	Saxifragaceae	H/D	8.3	23.2	2.82	0.426	0.848	62	-1.1	0.153
*Hibiscus moscheutos*	Malvaceae	H/D	9.3	14.2	1.58	0.268	0.813	25	6.0	0.026
*Hosta fortunei*	Asparagaceae	H/D	22.5	62.5	2.9	0.239	0.731	108	-9.3	0.072
*Ipomoea purpurea*	Convolvulaceae	H/D	4.8	17.5	3.58	0.196	0.781	39	-4.2	0.012
*Menyanthes trifoliata*	Menyanthaceae	H/D	6.6	19.3	2.96	0.285	0.851	53	-1.5	0.037
*Mirabilis longiflora*	Nyctaginaceae	H/D	4.4	11.4	2.57	0.351	0.841	28	-0.2	0.043
*Paeonia potaninii*	Paeoniaceae	H/D	11.7	37.3	3.19	0.431	0.887	131	-3.6	0.242
*Persicaria amplexicaulis*	Polygonaceae	H/D	5.8	15.4	2.71	0.243	0.705	25	-2.5	0.018
*Phytolacca americana*	Phytolaccaceae	H/D	7.2	20.0	2.8	0.232	0.846	53	-1.0	0.022
*Talinum paniculatum*	Talinaceae	H/D	2.8	7.3	2.65	0.973	0.923	36	0.2	0.581
*Viola sororia*	Violaceae	H/D	14.4	46.1	3.34	0.175	0.654	71	-14.3	0.020
*Epimedium versicolor*	Berberidaceae	H/E	52.7	151.4	2.87	0.177	0.808	338	-15.6	0.068
*Helleborus orientalis*	Ranunculaceae	H/E	54.6	158.1	2.91	0.304	0.8	345	-17.2	0.381
*Calycanthus occidentalis*	Calycanthaceae	W/D	24.9	61.3	2.47	0.26	0.839	149	1.3	0.090
*Cornus sanguinea*	Cornaceae	W/D	6.7	14.6	2.19	0.194	0.837	34	1.5	0.009
*Cornus stolonifera*	Cornaceae	W/D	10.5	19.9	1.85	0.185	0.842	43	4.5	0.012
*Diospyros virginiana*	Ebenaceae	W/D	20.3	57.9	2.86	0.252	0.849	157	-3.6	0.078
*Erythrina crista-galli*	Fabaceae	W/D	34.0	84.1	2.53	0.321	0.866	232	2.8	0.238
*Euonymus hamiltonianus*	Celastraceae	W/D	16.2	39.7	2.47	0.302	0.825	91	0.5	0.093
*Fagus sylvatica*	Fagaceae	W/D	56.2	141.5	2.57	0.159	0.852	368	2.2	0.048
*Ficus carica*	Moraceae	W/D	12.7	17.1	1.43	0.329	0.769	23	9.5	0.050
*Hydrangea macrophylla*	Hydrangeaceae	W/D	7.7	17.1	2.23	0.404	0.883	51	2.0	0.093
*Magnolia salicifolia*	Magnoliaceae	W/D	18.3	45.2	2.66	0.179	0.838	104	0.4	0.021
*Parrotiopsis jacquemontiana*	Hamamelidaceae	W/D	31.5	69.3	2.21	0.221	0.831	161	7.0	0.060
*Populus tremula*	Salicaceae	W/D	76.9	138.9	1.86	0.183	0.813	258	34.4	0.074
*Arbutus unedo*	Ericaceae	W/E	31.1	90.8	2.94	0.359	0.851	253	-6.7	0.354
*Aucuba japonica*	Garryaceae	W/E	26.7	75.1	2.84	0.344	0.865	217	-3.6	0.247
*Camellia japonica*	Theaceae	W/E	30.9	70.8	2.29	0.541	0.901	247	7.7	0.945
*Eucalyptus pauciflora*	Myrtaceae	W/E	83.6	176.2	2.15	0.585	0.869	463	26.5	2.925
*Hedera helix*	Araliaceae	W/E	28.5	68.2	2.41	0.378	0.901	238	5.2	0.296
*Ilex aquifolium*	Aquifoliaceae	W/E	33.7	90.0	2.71	0.549	0.902	338	0.8	1.221
*Rhododendron catawbiense*	Ericaceae	W/E	38.1	91.0	2.41	0.378	0.888	298	6.6	0.408
*Sarcococca hookeriana*	Buxaceae	W/E	19.0	63.6	3.33	0.429	0.903	264	-6.8	0.401
*Skimmia japonica*	Rutaceae	W/E	19.7	61.1	3.1	0.506	0.912	266	-4.0	0.680

### Mechanical tests

From each sampled leaf, three intercostal lamina strips in total were excised avoiding primary veins (and also secondary veins when they were very distinct) by a pair of razor blades that were fixed in parallel and 0.52cm apart. These samples were then used for the following mechanical tests; one strip was used for the tensile test, and the other two for the bending test on both the adaxial and abaxial sides of the leaves. Each lamina strip was excised just before the measurement so that the test specimen was kept as fresh as possible. When leaves were too small to excise enough strips, adjacent leaves were also used. There were five replicates per species for each measurement. One set of replicates (tensile and bending tests of 36 species) was completed in one day. In total five consecutive days were required to complete the measurements of five replications of 36 species. Neither apparent deterioration nor systematic change in leaf mechanical properties was observed during the measurement period.

The tensile and bending tests were conducted with a general testing machine (5542, INSTRON, Canton, MA, USA) at room temperature (~23°C) and relative humidity (50–70%). In the tensile tests, the specimens were clamped by a pair of pneumatically controlled grips, and the free length between the clamps was ~5cm. Before the measurements, width (*w*) and free length (*l*_0_) of the strips were measured by a caliper and the thickness at the middle of the strips (*d*) was measured by a thickness gage (7313, Mitsutoyo, Japan). Tension was applied at a constant speed of 25mm min^-1^. Force (*F*, N) and displacement (*δ,* mm) were measured every 100 milliseconds until the specimen was torn (Supplementary Fig. S2). The Young’s modulus in the tensile tests (*E*_T_) was estimated from the initial slope of the relationship between force (*F*) and displacement (*δ*) based on the following relationship (Gere and Timoschenko, 1999) (Fig. 1):

$$F=E_{T}A\frac{\delta }{l_{0}}$$ (9)

where *A* is the cross-section area of the specimen and *l*_o_ is the original length of the specimen between the clamps. Tensile strength (the maximum force per unit cross-section area of the specimen) was also recorded.

In the bending tests, a leaf strip was placed on two supports 15mm apart, and force was applied at the middle of the specimen at a speed of 25mm min^-1^. Force (*F*) and displacement (*δ*) were measured as in the tensile test (Supplementary Fig. S2). The Young’s modulus in the bending test (*E*_B_) was estimated from the initial slope of the relationship between force and displacement based on the following relationship (Gere and Timoschenko, 1999) (Fig. 1):

$$\begin{array}{l} F=\frac{48E_{B}I}{L^{3}}\delta \end{array}$$ (10)

where *L* is the span length and *I* is the second moment of area of the specimen. In a specimen with a rectangular cross-section, *I* is typically calculated as 1/12*wd* ^3^, where *w* is width and *d* is thickness of specimen. The span-thickness ratio was reasonably high (53±20), thus shear deformation was negligible. Adaxial *E*_B_ and abaxial *E*_B_ measures were strongly correlated with each other (R^2^=0.97) with a slope close to 1 (slope=1.024, 95% confidence interval of the slope=0.95–1.10, standardised major axis slope; Warton *et al.*, 2006). Therefore adaxial *E*_B_ and abaxial *E*_B_ were combined and averaged per each leaf sample, and used for the calculations of *E*_f_ and *E*_c_.

### Anatomy analysis

Small segments (∼1×2mm) of lamina were excised for anatomical analyses from all measured leaves (*n*=5 for each species). The segments were fixed with 2.5% glutaraldehyde in 100mM phosphate buffer (pH=7.0) and kept at 4°C until analysis. Leaf segments were sliced at a thickness of ∼10 μm and stained with Nile blue, a lipophilic dye. UV fluorescence images of the section were taken with a microscope (AX-LH 100 Olympus Optical, Japan) and used to measure thicknesses of each layer (epidermis, palisade, spongy parenchyma, cuticle, and cell walls of the epidermis) with image analysis software (analySIS, Olympus, Japan). Intercellular airspace was calculated from leaf fresh mass, dry mass and thickness with an assumption that specific gravity of water and leaf solid tissue were 1 and 1.5g cm^-3^ respectively (Roderick *et al.*, 1999).

### Calculation and statistical analysis

The Young’s moduli of the epidermis layers (*E*_f_) and mesophyll layer (*E*_c_) were calculated with equations 7 and 8. In this study, ‘epidermis layer’ means epidermis tissues plus cuticle membranes, and ‘mesophyll layer’ means intervening tissues between the epidermis layers (mostly mesophyll cells and some minor veins). Data were log-transformed before the statistical analysis when the data were not normally distributed (Shapiro-Wilk test, P<0.05). Differences in the values among functional groups were tested with ANOVA. Multiple comparisons were done with a Bonferroni correction. Pearson’s test was used to test correlation (*r*) among traits. Standardized major axis slope (Warton *et al.*, 2006) was fitted to bivariate trait relationships. A phylogenetic tree was constructed for all measured species from the APG III (2009) with a program, Phylomatic (http://phylodiversity.net/phylomatic/) (see Supplementary Fig. S1). The branch length was assumed to be a constant since detailed divergence time for each species was not clear. Moran’s autocorrelation index was used to test whether there was a phylogenetic autocorrelation in traits (Moran, 1950; Paradis, 2012). Phylogenetically independent contrasts (PICs; Felsenstein, 1985) were used to test whether correlations among traits in the cross-species comparisons were driven by coordinated evolutionary trait-shifts in a convergent manner across the phylogeny. All statistical analyses were performed with the R software package (v3.0.1, R Foundation for Statistical Computing, Vienna, Austria).

## Results

**Fig. 3.**
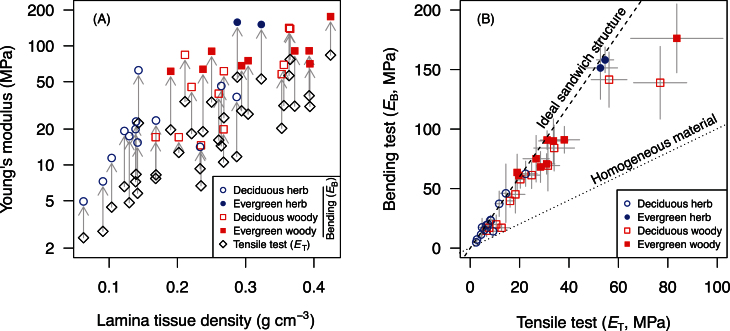
Leaf lamina Young’s modulus measured by bending tests (*E*_B_) and tensile tests (*E*_T_) for 36 angiosperm species. (A) *E*_B_ and *E*_T_ are plotted against lamina tissue density. The extent to which *E*_B_ values were higher than *E*_T_ is indicated by arrows. (B) Relationship between *E*_B_ and *E*_T_ across 36 angiosperm species. If the test piece is homogeneous, data should fall on 1:1 dotted line. Many species values fell close to the theoretical maximum 3:1 dashed line that is predicted by $\frac{E_{B}}{E_{T}}=\frac{1-\alpha^{\text{3}}\text{(1}-\beta \text{)}}{1-\alpha \text{(1}-\beta \text{)}}$ when *α*≈1, *β*≈0 (*α*, the fraction of mesophyll layer in leaf lamina; *β*, the ratio of the mesophyll layer Young’s modulus to the epidermis layer Young’s modulus), indicating that many leaf laminas had a nearly ideal sandwich structure. Mean and SD (for B) are shown for each species (*n*=5). (This figure is available in colour at *JXB* online.)

The Young’s moduli measured by tensile tests (*E*_T_) or bending tests (*E*_B_) were positively correlated to lamina tissue density, meaning that stiff leaves were made of dense tissues (Fig. 3A). The Young’s moduli measured by bending tests (*E*_B_) were consistently higher than *E*_T_ across 36 species, meaning that not only tissue density but also tissue arrangement, i.e. sandwich structure, affect the Young’s modulus when it is measured in bending (Fig. 3A). There was a strong correlation between *E*_T_ and *E*_B_ across species (R^2^=0.93, *P*<0.0001, Fig. 3B). The relationship was clearly different from the 1:1 relationship (*E*_B_/*E*_T_=2.59±0.48, *n*=36) and close to the theoretical maximum (*E*_B_/*E*_T_=3), meaning that leaf laminas of these plant species behaved as nearly ideal sandwich structures. Among growth forms, leaves of evergreen species were significantly stiffer (both *E*_T_ and *E*_B_) than those of deciduous species. The *E*_B_/*E*_T_ ratio was 2.77 in deciduous herbaceous species, 2.89 in evergreen herbaceous species, 2.28 in deciduous woody species and 2.67 in evergreen woody species.

**Fig. 4.**
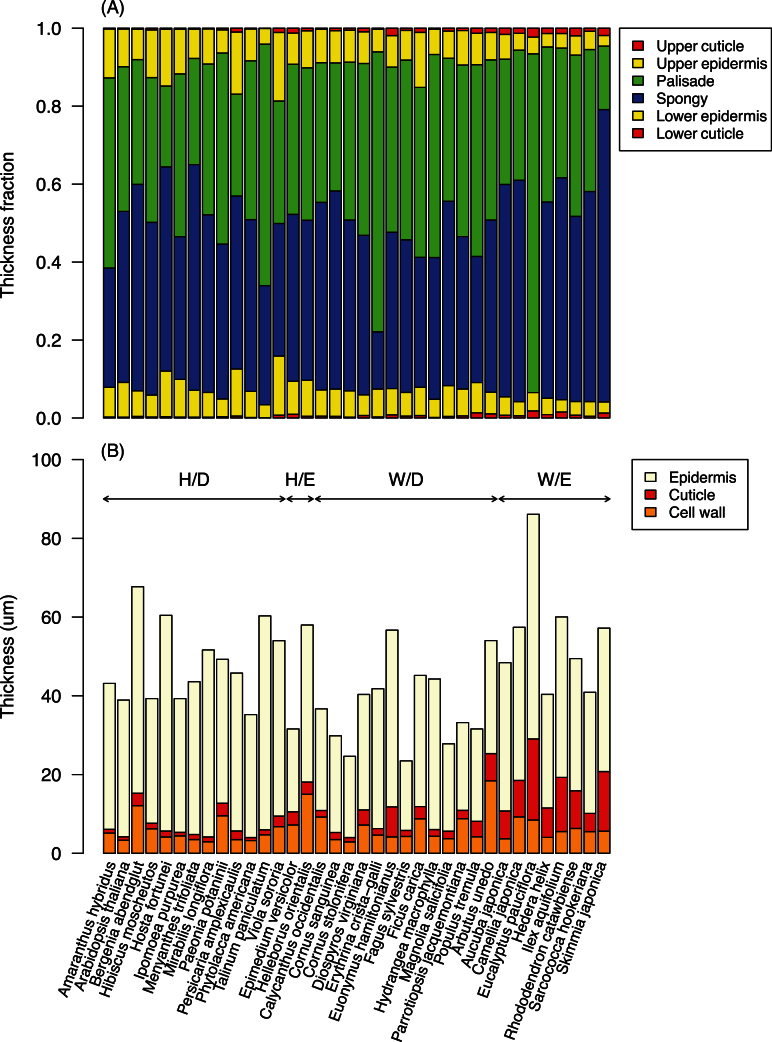
Leaf anatomy characteristics of 36 angiosperm species. (A) Thickness fraction of each layer. (B) Thickness of epidermis cell walls, cuticles and the rest of epidermis layer (upper and lower layers pooled together). Functional groups are abbreviated as H/D deciduous herbaceous species; H/E evergreen herbaceous species; W/D deciduous woody species; W/E evergreen woody species. Mean of five replications is shown for each species. (This figure is available in colour at *JXB* online.)

Lamina thickness varied from 0.159mm in *Fagus sylvatica* to 0.973mm in *Talinum paniculatum.* Among functional groups, deciduous herbaceous species had thicker laminas (0.33±0.21mm) than deciduous woody species (0.25±0.08mm) but thinner than evergreen woody species (0.45±0.09mm). The mean mesophyll thickness (expressed as a fraction of total lamina thickness) (*α*) across all 36 species was 83.6±5.8% (Table 1; Fig. 4A). Evergreen woody species had slightly but significantly higher *α* values (88.8±2.1%) than deciduous herbaceous species (80.5±7.4%) and deciduous woody species (83.7±2.8%). The number of species in evergreen deciduous species was small (two species) but the leaf thickness values were close to those of deciduous herbaceous species (Table 1).

**Table 2.** Cross-species correlations (Pearson’s test) and phylogenetic independent contrast (PIC) correlations among leaf traits across 36 species Abbreviations: E_f_ and E_c_ are Young’s modulus of epidermis and mesophyll layers respectively. E_T_ and E_B_ are Young’s modulus measured by the tensile test and bending test respectively. Log_10_-transformation was applied when data were not normally distributed (Trans = 1). Bold letters indicate the level of significance (*P*<0.05).

Traits	Trans	*E*_f_		*E*_c_	
		Cross-species	PIC	Cross-species	PIC
Lamina thickness	1	0.206	0.059	0.118	-0.049
Fresh LMA	1	0.265	0.139	0.126	-0.049
Dry LMA	1	**0.722**	**0.595**	0.263	0.13
Water content	0	**-0.735**	**-0.717**	-0.31	**-0.353**
Drymass density	0	**0.757**	**0.767**	0.316	**0.34**
Air fraction	0	0.008	-0.065	0.074	0.183
*E*_T_	1	**0.908**	**0.894**	0.239	**0.436**
*E*_B_	1	**0.958**	**0.964**	0.087	0.252
*E*_B_/*E*_T_	0	0.246	0.181	**-0.673**	**-0.723**
Tensile strength	0	**0.86**	**0.858**	0.054	0.279
Bending stiffness per unit width	1	**0.731**	**0.607**	0.151	0.111
Upper cuticle thickness	1	**0.653**	**0.447**	0.204	0.031
Upper epidermis thickness	0	**-0.397**	**-0.522**	-0.251	**-0.357**
Palisade thickness	1	0.274	0.244	0.281	0.161
Spongy thickness	1	0.224	0.196	-0.124	-0.213
Lower epidermis thickness	0	-0.096	-0.295	-0.245	**-0.387**
Lower cuticle thickness	1	**0.708**	**0.53**	0.22	0.121
Upper epidermis cell wall thickness	1	**0.354**	-0.09	-0.087	-0.184
Lower epidermis cell wall thickness	1	**0.477**	-0.079	-0.179	-0.276
Total epidermis thickness	0	-0.018	-0.272	-0.117	-0.351
Mesophyll fraction (*a*)	0	**0.398**	**0.626**	0.245	**0.386**
(Cuticle + cell wall)/epidermis	0	**0.762**	**0.632**	0.155	0.174
Leaf area	1	-0.18	-0.268	-0.065	-0.077
*E*_f_	1	---	---	0.069	0.196
*E*_c_	0	0.069	0.196	---	---

For the epidermis layer, evergreen species had more than 4-fold thicker cuticle layers (9.17±5.39 µm, *n*=11) than deciduous species (2.14±1.46 µm, *n*=25), while the epidermis cell wall thickness was only marginally higher in evergreen species (8.09±4.63 µm) than in deciduous species (5.39±2.49 µm) (Fig. 4B).

**Fig. 5.**
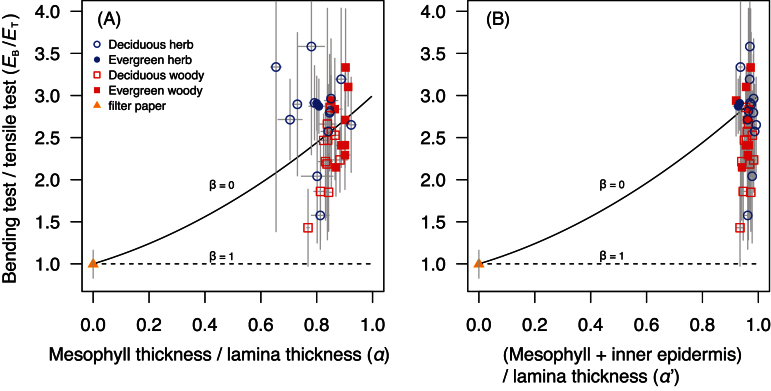
The ratios of the leaf lamina Young’s moduli measured by bending tests to those measured by tensile tests (*E*_B_/*E*_T_) are plotted against (A) α (the fraction of mesophyll layer in leaf lamina) and against (B) α‘ (the fraction of mesophyll layer plus inner epidermis layer in leaf lamina) across 36 angiosperm species. The lines predicted by $\frac{E_{B}}{E_{T}}=\frac{1-\alpha^{\text{3}}\text{(1}-\beta \text{)}}{1-\alpha \text{(1}-\beta \text{)}}$ are overlaid (*β* is the ratio of the mesophyll layer Young’s modulus to the epidermis layer Young’s modulus). Mean and SD are shown for each species (*n*=5). Filter papers were included in this comparison as an example of homogeneous material (*n*=12). (This figure is available in colour at *JXB* online.)

The relationship between *E*_B_/*E*_T_ and the mesophyll fraction (*α*) is shown in Fig. 5A. All species fell in the region of high *E*_B_/*E*_T_ ratio (>1) and high *α* (close 1) as expected for sandwich structures. However, there were some species with higher *E*_B_/*E*_T_ ratios than the theoretical maximum lines (1+*α*+*α* ^2^, see equation 6), which can be achieved when *β* (=*E*_c_/*E*_f_) equals 0. This deviation might be because (i) *α* was underestimated, (ii) there were some mechanisms beyond the linear elastic theory or (iii) other technical issues (see Discussion). When the inner part of epidermis layer was included in the mesophyll fraction (*α‘*), most species fell within the theoretical lines (Fig. 5B).

**Fig. 6.**
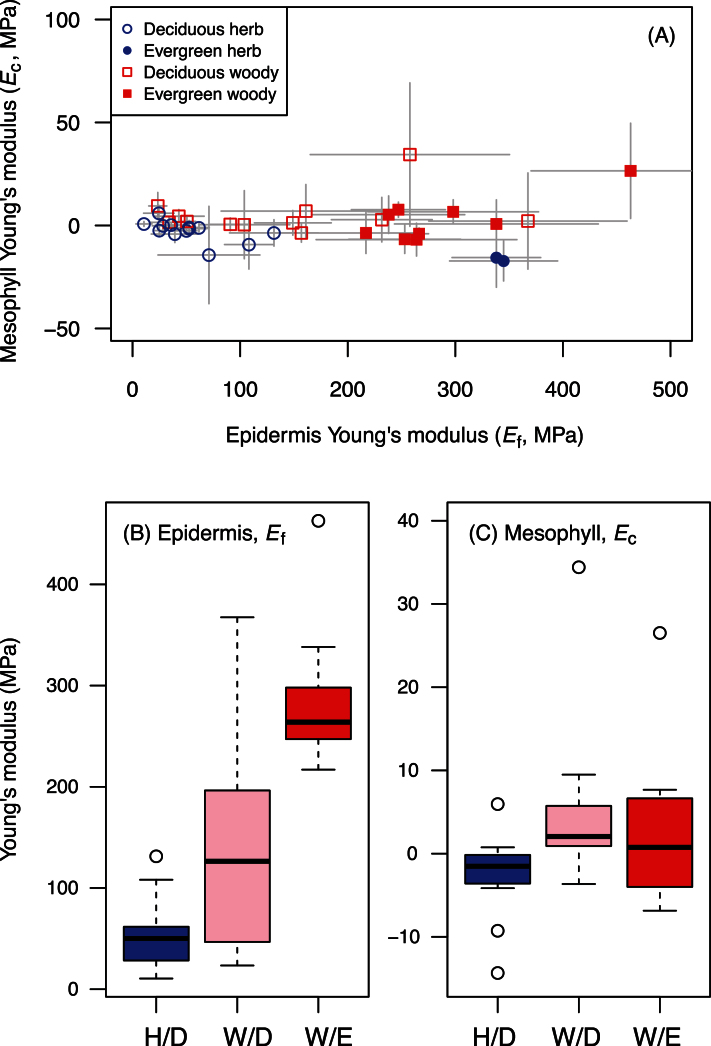
The Young’s moduli of the epidermis (*E*_f_) and mesophyll layers (*E*_c_) for 36 angiosperm species. (A) The relationship between the two moduli. Box plot for (B) *E*_f_ and for (C) *E*_c_ across three functional groups (H/D, deciduous herbaceous species; W/D deciduous woody species; W/E evergreen woody species). The central box in each box plot shows the interquartile range and median and whiskers indicate the 10th and 90th percentiles. For (B, C), evergreen herbaceous species are not included due to the small sample size (*n*=2). (This figure is available in colour at *JXB* online.)

The epidermis and mesophyll Young’s moduli (*E*_f_ and *E*_c_) were calculated by applying the measured values of *E*_B_, *E*_T_ and the mesophyll fraction (*α*) to equations 7 and 8. *E*_f_ was much higher than *E*_c_, ranging from 11MPa in *Arabidopsis thaliana* to 463MPa in *Eucalyptus pauciflora* (Table 1; Fig. 6). On the other hand, *E*_c_ was much lower (0.61±9.61MPa, *n*=36). The relatively large SD for the mean (i.e. high coefficient of variation) in *E*_c_ was partly due to the structure of the data. For example, *E*_c_ was calculated from *E*_B_ and *E*_T_ (equation 8) and therefore variation in *E*_c_ was comparable in magnitude to the variation of *E*_B_ and *E*_T_, whereas the mean value of *E*_c_ was much smaller than those of *E*_B_ and *E*_T_. While the accurate estimate of *E*_c_ might not be possible in this method (see Discussion), qualitatively it is evident that the longitudinal stiffness of leaf laminas was largely determined by the stiffness of the epidermis layers. Among functional groups, *E*_f_ was higher in evergreen woody species than in deciduous woody and herbaceous species (Fig. 6B, *P*<0.01).

**Fig. 7.**
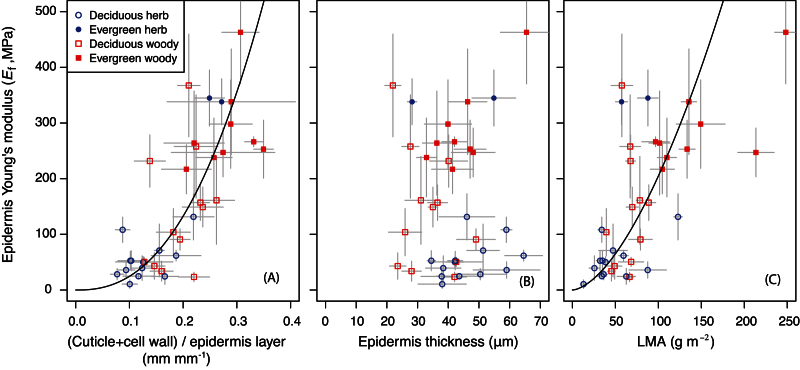
The Young’s moduli of the epidermis layers were plotted against (A) the fraction of cuticle and outer cell walls within the epidermis layers, (B) thickness of the epidermis layers (both upper and lower layers pooled together) and (C) leaf mass per area (LMA) across 36 angiosperm species. Mean and SD are shown for each species (*n*=5). Curvilinear regressions are used as they fit better than linear regressions. Regression lines; (A) y=6451x^2.41^, R^2^=0.56, P<0.001; (C) y=0.1004x^1.65^, R^2^=0.52, P<0.001 Note that ‘epidermis layer’ in this study includes epidermis tissues and cuticles. (This figure is available in colour at *JXB* online.)

The variation in the epidermis Young’s modulus (*E*_f_) was significantly correlated with the fraction of outer cell walls and cuticles within the epidermis layers (R^2^=0.56, *P*<0.001, *n*=36) (Fig. 7A). Each component, i.e. cuticle fraction or outer cell wall fraction in the epidermis layers, significantly correlated to the epidermis Young’s modulus (*P*<0.001), but the cuticle fraction rather than the cell wall fraction correlated more strongly with the epidermis Young’s modulus (R^2^=0.43–0.50 versus 0.13–0.23, Table 2). The thickness of the epidermis layer itself was not significantly correlated to the epidermis Young’s modulus (Fig. 7B, Table 2, *P*>0.05), while leaf mass per area (LMA) was significantly correlated to the epidermis Young’s modulus (Fig. 7C, Table 2, R^2^ = 0.52). The Young’s moduli of mesophyll layers were not significantly correlated with any leaf morphological or anatomical traits unless phylogeny was considered (Table 2). When phylogeny was considered, the Young’s moduli of mesophyll layers were slightly negatively correlated to water content and positively correlated to the mesophyll fraction.

Phylogenetic autocorrelations were weak and were not significant in most of the measured traits (Supplementary Table S2). Furthermore, a consideration of phylogenetic divergences did not strongly alter levels of correlations among traits (Table 2). The interpretation of these results is that the trait associations observed in this study were a result of repeated evolution across different clades of plants rather than a phylogenetic bias in the selection of studied species.

## Discussion

In this study, we developed and applied a novel method to estimate the Young’s moduli of epidermis and mesophyll layers. Our model based on linear elastic theory has a number of simplifying assumptions, and there were some phenomena that cannot be fully explained by this model (as discussed later). Yet, our approach showed that leaf laminas were designed as efficient sandwich structures in which the epidermis tissues were much stiffer and thinner than the mesophyll tissues. In addition, we showed that this sandwich structure was found in a wide range of studied angiosperm species, suggesting that a sandwich structure has general advantages for many land plants. In this discussion, we discuss anatomical and ecological considerations of leaf sandwich structures and also discuss future challenges including technical issues.

### Anatomical considerations

Across species, the epidermis Young’s modulus was strongly correlated with the fraction of cuticles and outer cell walls in the epidermis layer (Fig. 7), suggesting that the cuticles and epidermis outer cell walls played major roles in determining the surface stiffness. In particular, evergreen leaves had stiffer epidermises (Fig. 6) and thicker cuticles than deciduous leaves (Fig. 4), suggesting cuticles are mechanically important for long-lived leaves. This implication is also supported by studies on isolated leaf cuticle membranes (Wiedemann and Neinhuis, 1998; Bargel *et al.*, 2006; Onoda *et al.*, 2012), which found that leaf cuticles were made of very stiff material (50–1,500MPa). Furthermore *E*_f_ increased more than proportionally with the fraction of cuticles and outer cell walls in the epidermis layer, indicating that thicker cuticles were made of stiffer materials (Onoda *et al.*, 2012).

The mesophyll tissues were much softer than the epidermis tissues, which should be due to the thin mesophyll cell walls, little longitudinal continuity and the presence of intercellular airspaces. Mesophyll cells generally have much thinner cell walls (0.05–0.4 μm, Terashima *et al.*, 2011) than epidermis cells (1.4–9.2 μm, Fig. 4B). Mesophyll layers also contain larger intercellular airspaces; our calculation showed that 7–41% of the leaf volume was occupied by air (Supplementary Table S1). The thin cell walls of mesophyll and the presence of intercellular airspaces are primarily important to facilitate a high rate of CO_2_ diffusion for photosynthesis (Parkhurst, 1977; Terashima *et al.*, 2001), but our study suggests that air spaces can also contribute to higher bending stiffness (i.e., the product *E*_B_ *I*) by increasing the second moment of area (*I*) in the sandwich structure.

There were minor veins in our test specimens, but their contribution to bending stiffness were likely small, as indicated by low *E*_c_. This was in part due to their location close to the neutral axis, i.e. under bending, minor veins located close to the neutral axis deform much less than outer tissues (i.e. epidermis). Thus minor veins geometrically cannot contribute much to *E*_B_. Such negligible contribution of minor veins to maintain a leaf horizontal position was also reported by a finite element analysis (Kobayashi *et al.*, 2000, 2002). Studies on hypocotyls of *Helianthus annuus* (Asteraceae) also showed that 86% of tensile stiffness came from the epidermis (Hejnowicz and Sievers, 1995*b*). Similarly, in turgid tulip stems the epidermis was found to contribute up to 50% of stem bending stiffness (Niklas and Paolillo, 1997). Minor leaf veins are however important for water and photosynthate transport (Brodribb *et al.*, 2007; Sack *et al.*, 2013) and shear resistance (Lucas *et al.*, 1991).

### Ecological significance

Some leaves, especially herbaceous ones, are turgid i.e. hydrostatically inflated. They rely on turgor pressure to increase bending stiffness without much investment of resources in cell walls. It was intriguing that herbaceous species had significantly higher *E*_B_/*E*_T_ values than woody species (2.80±0.48 versus 2.45±0.44, t-test, *P*<0.05) as well as higher water content (0.79±0.10 versus 0.64±0.07, t-test, *P*<0.05), suggesting that turgor pressure can increase efficiency of the sandwich structure. In turgid leaves, epidermis and mesophyll layers are maintained in states of tension and compression respectively because the hydrostatically inflated ‘core’ mesophyll is accommodated by the much stiffer ‘face’ epidermis layers (Hejnowicz and Sievers, 1995*a*; Kutschera and Niklas 2007). This ‘pre-stressed sandwich structure’ may increase efficiency of sandwich structure in at least two ways. First, the hydrostatic pressure increases lamina thickness and thus widens the distance between two epidermis layers. This increases the second moment of area of the epidermis layers in relation to the neutral axis and contributes to more efficient sandwich structure. Second, the epidermis tissue may be ‘strain-stiffening material’ (Hejnowicz and Sievers, 1996). While this phenomenon cannot be explained by the linear elastic theory, if the epidermis layers become stiffer under hydrostatic pressure, they can increase efficiency of a sandwich structure by decreasing *β* (=*E*_c_/*E*_f_). Such mechanical efficiency should enable herbaceous plants to maintain a larger leaf area for a given biomass, which could contribute to them having relatively fast growth rates compared to woody species (Poorter *et al.*, 2009), although other factors play a role as well. On the other hand, due to their reliance on water, hydrostatically inflated leaves tend to wilt sooner than leaves of woody species when water is limited. Leaf wilting is also an important function for plants as they can avoid strong sunlight and reduce water loss from leaves. Thus, the hydrostatically controlled-sandwich structure may be a quite efficient mechanism enabling plants to both acquire and avoid light energy depending on water availability.

In terms of leaf mechanical stability, a sandwich structure may be particularly beneficial for plants growing under light limited conditions because they are often carbon-limited and produce thinner leaves (as compared to the same species growing under stronger light) to achieve high efficiency of light interception per unit biomass (Terashima *et al.*, 2001; Poorter *et al.*, 2009). These shaded leaves tend to have a larger fraction of intercellular airspace (e.g. Lee *et al.*, 2000). Indeed in *Plantago major*, the fraction of intercellular airspace was found to increase by 28% with a shading treatment (Onoda *et al.*, 2008). The relatively large airspace in shade leaves was more likely associated with demands on mechanical stability than with the necessity for rapid intracellular CO_2_ diffusion for photosynthesis, since photosynthesis under shade is hardly limited by the intercellular CO_2_ diffusion resistance (Terashima *et al.*, 2001). Bending stiffness scales to the third power of lamina thickness and, for example, a 10% larger lamina thickness due to an increase intercellular space can increase lamina bending stiffness by 33% without adding biomass. Therefore a larger intercellular space is likely important to maintain larger light interception area for a given mass which is advantageous in shaded environments.

Overall we observed a large interspecific variation in leaf lamina stiffness indices i.e. 36-fold in *E*_B_ and 790-fold in bending stiffness (= *E*_B_ × *I*) (Table 1), while the interspecific variation in fresh leaf mass per unit leaf area was much smaller (7-fold) (Supplementary Table S1). The much larger variation in bending stiffness than in fresh leaf mass may suggest that some leaves (especially evergreen leaves) may be too stiff (over-engineered) relative to the need to resist gravity or moderate wind, although an integrative approach including leaf weight, material properties and leaf dimensions is needed to understand whole leaf elastic stability (e.g. Fournier *et al.*, 2013; Tadrist *et al.*, 2014). These leaves were nevertheless designed as sandwich structures as indicated by their high *E*_B_/*E*_T_ ratios, suggesting that a sandwich structure has benefits beyond being efficient in resisting gravitational and wind forces. Relatively thick cuticles may act as stiff and tough barriers against attacks from herbivores and microorganisms e.g. pathogens and bacteria (Grubb, 1986), and leaf toughness is a key element for longer leaf lifespan (Coley, 1983; Kitajima and Poorter, 2010; Onoda *et al.*, 2011). Actually, in the present study, the Young’s moduli of the epidermis layers were strongly correlated with LMA (leaf mass per area), which is a good indicator of leaf lifespan (Wright *et al.*, 2004). These observations suggest that sandwich structures may be beneficial not only for maintaining leaf plane structure against gravity or moderate wind, but also for long lifespan by protecting leaf functions from various external stresses.

While bending stiffness is important for leaves, leaves should be also deformable to reduce drag during strong winds (Vogel, 1989). This requirement may sound contradictory with the requirement for stiffness, but it may be possible because leaf surfaces (i.e. cuticles) are highly extensible materials in spite of their stiffness (Onoda *et al.*, 2012). The requirement for deformation is likely to increase with leaf size because drag force is proportional to surface area (Vogel, 1989). In other words, small leaves do not necessarily deform much under wind. This may accord with the general pattern that very stiff leaves (e.g. sclerophyll leaves) tend to be small (Grubb, 1986).

### Technical issues and future challenges

In this study, we show that the *E*_B_/*E*_T_ ratio is a useful indicator of the extent to which leaves are designed as sandwich structures. Our method seems to be applicable to plant leaves since the *E*_B_/*E*_T_ ratio of filter papers, which were more or less homogeneous material, was close to 1 (0.996±0.17) while most leaves had the ratios much higher than 1 (2.59±0.48, n=36). However, there were some species that exceeded the theoretical threshold of *E*_B_/*E*_T_ (=3), albeit by a relatively small margin. Similarly, some mesophyll tissues had slightly negative Young’s moduli while the Young’s modulus should be a positive value. These may be artifacts possibly resulting from assumptions made in this study. We have at least three possible explanations for these phenomena. (i) The *effective* thickness of the stiff epidermis layer may be smaller than the total thickness of the epidermis layer. This is because the cuticle and *outer* cell walls largely determine the epidermis Young’s modulus (Fig. 7), whereas the inner part of the epidermis may behave more like the mesophyll tissues (Fig. 5B). If so, our mesophyll Young’s modulus values were underestimations and this could have resulted in negative values. Actually, recalculating the Young’s modulus of the mesophyll layer in this way, two-thirds of species that initially had negative moduli switched to positive values. However, this issue cannot explain why some leaves had *E*_B_/*E*_T_ values larger than 3 (Fig. 5). (ii) There was a possible technical issue with the measurement. If leaf laminas were not completely flat in a direction perpendicular to the longitudinal direction (although we tried to exclude non-flat leaves), the effective second moment of area could be higher than the calculated second moment of area that assumed a rectangular cross-section. If so, this would result in an over-estimation of *E*_B_ while *E*_T_ would be unaffected. Similarly, if lamina thickness was not homogeneous within a leaf specimen, a slight underestimation of lamina thickness might result in an over-estimation of *E* and this effect would have been larger in the bending test than in the tensile test. These technical issues can result *E*_B_/*E*_T_ values >3 and, subsequently, an apparent negative mesophyll Young’s modulus. (iii) As mentioned earlier, biological materials are quite complicated and do not always behave according to the linear elastic theory. It is known that soft tissues like mesophyll behave like ‘Fung materials’, which have a larger resistance to compressive strain than to tensile strain (Fung, 1993; Vandiver and Goriely, 2008). Furthermore, the epidermis tissue may be ‘strain-stiffening material’ (Hejnowicz and Sievers, 1996). There are also other effects including visco-elasticity, interactions between stress and moisture content, geometric non-linearity produced, e.g. by the Poisson’s effect (Moulia, 2013). These effects together may lead to *E*_B_/*E*_T_>3 and, consequently, result in apparent negative mesophyll Young’s moduli. While our aim was to understand the basic principle underlying the leaf mechanical design across species, these complicating factors cannot be ignored and could be important for mechanical stability of leaves.

In this study, we focused on leaf laminas and did not consider mechanical properties of major veins or petioles. The mechanical contribution of major veins to whole leaf bending stiffness is important, especially near the leaf base, while lamina stiffness has a major role in leaf bending stiffness toward the leaf tips (Moulia *et al.*, 1994; Kobayashi *et al.*, 2000, 2002). Integrating the geometry and mechanical properties of laminas, veins and petioles will be an important challenge to understand mechanical stability of whole leaves (Tadrist *et al.*, 2014).

### Conclusions

Our novel approach indicates that many leaf laminas are designed as ideal sandwich structures, with the exclusively thin and stiff epidermis layers enveloping a thicker and softer mesophyll core. We showed that this design principle is commonly found in broad-leaves across a wide range of angiosperm taxa. This structure enables land plants to produce thin flat leaves with a high light interception per unit invested mass and yet resistant to buckling under gravitational forces and moderate wind. The stiff surface should also be important as a barrier against external biotic and abiotic stresses such as herbivory, pathogen attacks and rainfall. Anatomical features such as stiff cuticles, thin mesophyll cell walls, large intercellular airspace and hydrostatic pressure within leaves seem to be elegantly coordinated to optimize multi-functions of leaves, such as photosynthesis, mechanical stability and defence. These results provide new insights into the functional significance of leaf structure and have implications for plant sciences and biomimetic studies.

## Supplementary material

Supplementary material is available at *JXB* online.

Supplementary Text S1. A full derivation of equations 7 and 8.

Supplementary Table S1. Additional leaf traits of 36 species.

Supplementary Table S2. Phylogenetic autocorrelations for measured traits across 36 species.

Supplementary Fig. S1. A phylogenetic tree of 36 species.

Supplementary Data
