# Supplementary material for: A novel method of measuring leaf epidermis and mesophyll stiffness shows the ubiquitous nature of the sandwich structure of leaf laminas in broad-leaved angiosperm species
Source: J Exp Bot. 2015 Feb 11;66(9):2487–99. doi: 10.1093/jxb/erv024 (PMC4986859; doi:10.1093/jxb/erv024)
Supplement: Supplementary Data [file supp_66_9_2487__index.html]

A novel method of measuring leaf epidermis and mesophyll stiffness shows the ubiquitous nature of the sandwich structure of leaf laminas in broad-leaved angiosperm species — Supplementary Data 

# A novel method of measuring leaf epidermis and mesophyll stiffness shows the ubiquitous nature of the sandwich structure of leaf laminas in broad-leaved angiosperm species

## Supplementary Data

Data files

**Files in this Data Supplement:**

- Supplementary Data - Supplementary Data
