# Supplementary material for: A novel method of measuring leaf epidermis and mesophyll stiffness shows the ubiquitous nature of the sandwich structure of leaf laminas in broad-leaved angiosperm species
Source: J Exp Bot. 2015 Feb 11;66(9):2487–99. doi: 10.1093/jxb/erv024 (PMC4986859; doi:10.1093/jxb/erv024)
Supplement: Supplementary Data [file supp_erv024_Onoda_SI.pdf]

Supporting information

Text S1. A full derivation of Eq. 7 and 8 from the Eq. 3 and 4 in the main text is shown below. Eq. 3 and 4 are expressed as follows:

$$E_T = (1-\alpha)E_f + \alpha E_c \qquad \text{Eq. 3}$$

$$E_B = (1-\alpha^3)E_f + \alpha^3 E_c \qquad \text{Eq. 4}$$

In these equations, there are only two variables that cannot be directly measured (i.e. E_c and E_f), thus these two variables can be mathematically solved. Reformulating Eq. 3 for E_c gives;

$$E_c = (E_T - (1-\alpha)E_f)/\alpha \qquad \text{Eq. S1}$$

Applying this equation to Eq.4 yields;

$$\begin{aligned} E_B &= (1-\alpha^3)E_f + \alpha^2(E_T - (1-\alpha)E_f) \\ &= (1-\alpha^2)E_f + \alpha^2 E_T \end{aligned} \qquad \text{Eq. S2}$$

Therefore E_f is solved as;

$$E_f = \frac{E_B - \alpha^2 E_T}{1-\alpha^2} \qquad \text{Eq. 7}$$

In a similar manner, by applying Eq. 7 to Eq. S1, E_c is solved as;

$$E_c = \frac{(1+\alpha+\alpha^2)E_T - E_B}{\alpha(1+\alpha)} \qquad \text{Eq. 8}$$

Table S1. Abaxial- and adaxial Young's moduli measured by bending tests (E_B, MPa) and fresh leaf mass per area (fLMA, g m^{-2}), dry leaf mass per area (LMA, g m^{-2}), water content (Water, g g^{-1}), estimated volume fraction of leaf occupied by air (Air, m^3 m^{-3}), and leaf area (LA, m^2) of 36 species. Mean of 5 replications.

Species	E_B adaxial	E_B abaxial	fLMA	LMA	Water	Air	LA
Amaranthus hybridus	24.8	22.5	187	38.6	0.794	0.237	66
Arabidopsis thaliana	6.9	3.0	158	12.9	0.919	0.256	7.1
Bergenia abendglut	23.8	22.5	338	59.5	0.824	0.253	16.7
Hibiscus moscheutos	13.8	14.6	203	62.7	0.694	0.313	61.7
Hosta fortunei	59.6	65.4	188	34.2	0.818	0.262	232.7
Ipomoea purpurea	14.9	20.1	172	25.6	0.852	0.172	58
Menyanthes trifoliata	21.3	17.4	194	35	0.819	0.362	28.4
Mirabilis longiflora	10.6	12.3	274	36.4	0.867	0.254	52.5
Paeonia potaninii	39.6	35.0	376	122.9	0.672	0.221	45.5
Persicaria amplexicaulis	12.9	18.0	155	34.3	0.779	0.406	39.1
Phytolacca americana	19.6	20.5	210	32.2	0.874	0.139	29.3
Talinum paniculatum	7.3	7.3	935	87.6	0.905	0.071	30.7
Viola sororia	52.5	39.6	156	47.2	0.704	0.188	29.6
Epimedium versicolor	154.8	148.0	144	57.2	0.605	0.292	59.9
Helleborus orientalis	161.6	154.6	258	87.9	0.66	0.249	34.6
Calycanthus occidentalis	60.6	62.0	199	69.7	0.651	0.322	121
Cornus sanguinea	12.9	16.3	142	45.4	0.679	0.347	57.1
Cornus stolonifera	22.6	17.2	133	49.1	0.633	0.361	50.1
Diospyros virginiana	63.3	52.5	231	88.8	0.616	0.198	44.5
Erythrina crista-galli	82.3	85.8	261	67.7	0.741	0.256	192.1
Euonymus hamiltonianus	41.0	38.4	257	79.2	0.693	0.239	69.1
Fagus sylvestris	138.9	144.1	129	57.5	0.552	0.308	16.7
Ficus carica	17.1	17.1	220	66.5	0.697	0.399	56.1
Hydrangea macrophylla	18.2	16.1	310	68.3	0.782	0.287	129.8
Magnolia salicifolia	53.9	36.4	148	39.5	0.733	0.246	21.6
Parrotiopsis	75.8	62.9	182	78.5	0.57	0.292	25.3

jacquemontiana							
Populus tremula	148.0	129.9	156	67.2	0.569	0.275	39.9
Arbutus unedo	100.2	81.3	307	133.5	0.565	0.27	51.3
Aucuba japonica	75.7	74.5	291	104.7	0.641	0.255	81.1
Camellia japonica	65.7	75.8	497	213.6	0.571	0.213	28.1
Eucalyptus pauciflora	163.3	189.1	540	248.3	0.54	0.219	27
Hedera helix	70.9	65.4	307	109.9	0.643	0.277	31.2
Ilex aquifolium	90.3	89.8	383	135.6	0.646	0.378	10.1
Rhododendron catawbiense	98.8	83.2	324	149.2	0.54	0.276	21.3
Sarcococca hookeriana	67.3	59.8	323	101.4	0.685	0.326	33.6
Skimmia japonica	59.6	62.6	341	96.7	0.718	0.387	45.4

Table S2. Phylogenetic autocorrelations tested by Moran's I. See Table 2 for abbreviations.

Traits	Observed	Expected	SD	P-value
Lamina thickness	-0.019	-0.029	0.068	0.891
fresh LMA	-0.008	-0.029	0.067	0.764
dry LMA	0.009	-0.029	0.068	0.579
water content	0.122	-0.029	0.069	0.030
drymass density	0.119	-0.029	0.069	0.034
Air fraction	0.027	-0.029	0.068	0.415
E_T	0.137	-0.029	0.069	0.016
E_B	0.146	-0.029	0.069	0.011
E_B/E_T	0.022	-0.029	0.068	0.455
Tensile strength	0.181	-0.029	0.069	0.002
Bending stiffness per unit width	-0.006	-0.029	0.069	0.748
Upper cuticle thickness	0.074	-0.029	0.069	0.138
Upper epidermis thickness	-0.054	-0.029	0.069	0.709
Palisade thickness	-0.041	-0.029	0.067	0.854
Spongy thickness	0.091	-0.029	0.072	0.098
Lower epidermis thickness	-0.076	-0.029	0.069	0.486
Lower cuticle thickness	0.068	-0.029	0.068	0.160
Upper epidermis cell wall thickness	0.135	-0.029	0.068	0.017
Lower epidermis cell wall thickness	0.148	-0.029	0.069	0.010
Total epidermis thickness	-0.058	-0.029	0.068	0.667
Mesophyll fraction (a)	0.016	-0.029	0.066	0.505
(Cuticle + cell wall)/epidermis	0.150	-0.029	0.069	0.010
Leaf area	0.019	-0.029	0.068	0.479
E_f	0.089	-0.029	0.069	0.089
E_c	-0.001	-0.029	0.064	0.666

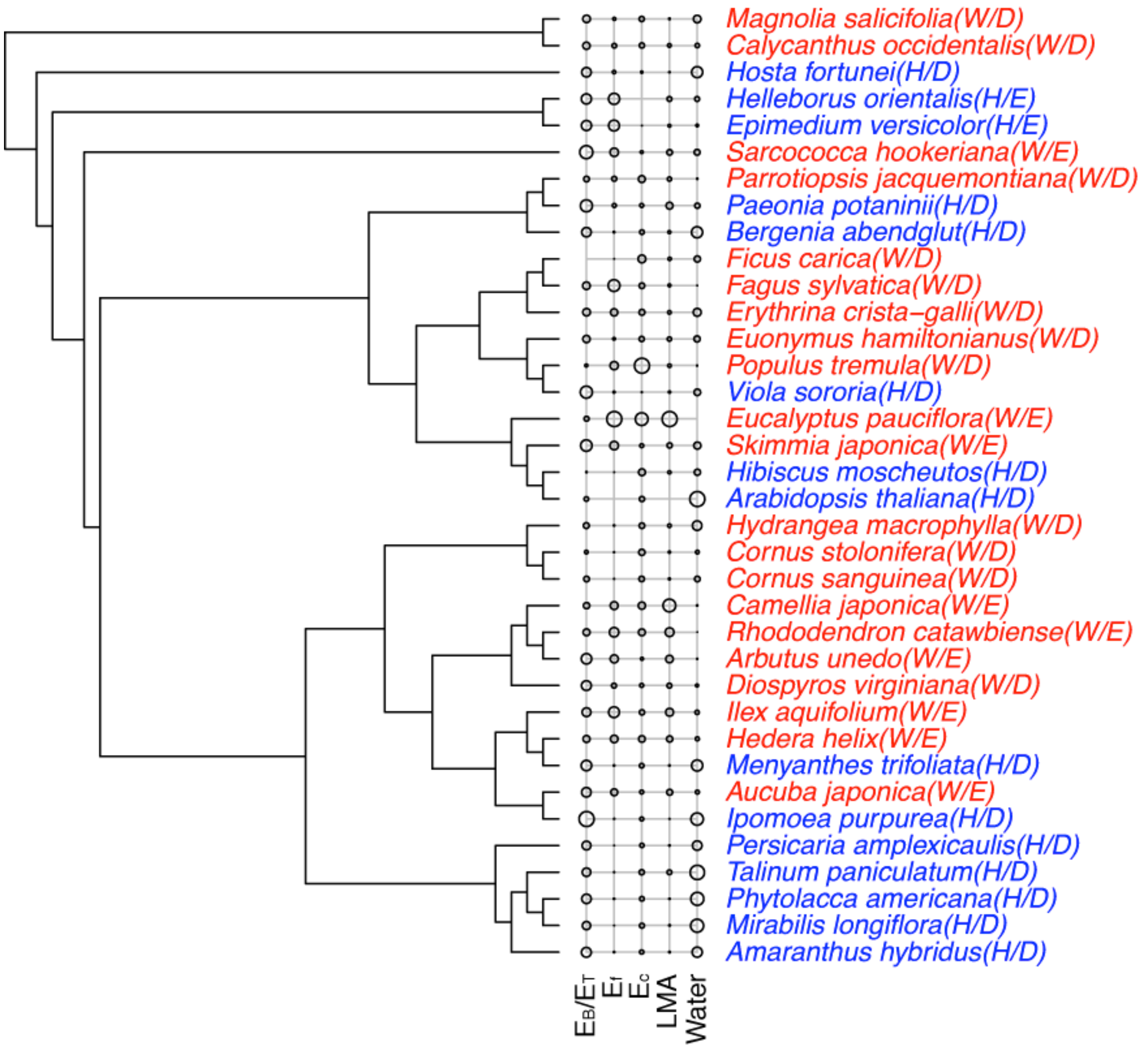

Fig. S1. A phylogenetic tree and five key traits of studied species. Species names in red denote woody species (W) and those in blue denotes herbaceous species (H). D and E in the parentheses denote deciduous and evergreen leaf habits respectively. Trait values are shown as the size of circles (i.e. larger circles correspond to large values). E_B/E_T is an indicator of level of sandwich structures evaluated as the ratio of Young's moduli measured by a bending test to that by a tensile test. E_f, Young's modulus of epidermis layer; E_c, Young's modulus of mesophyll layer; LMA, leaf mass per area; Water, leaf water content per unit fresh mass.

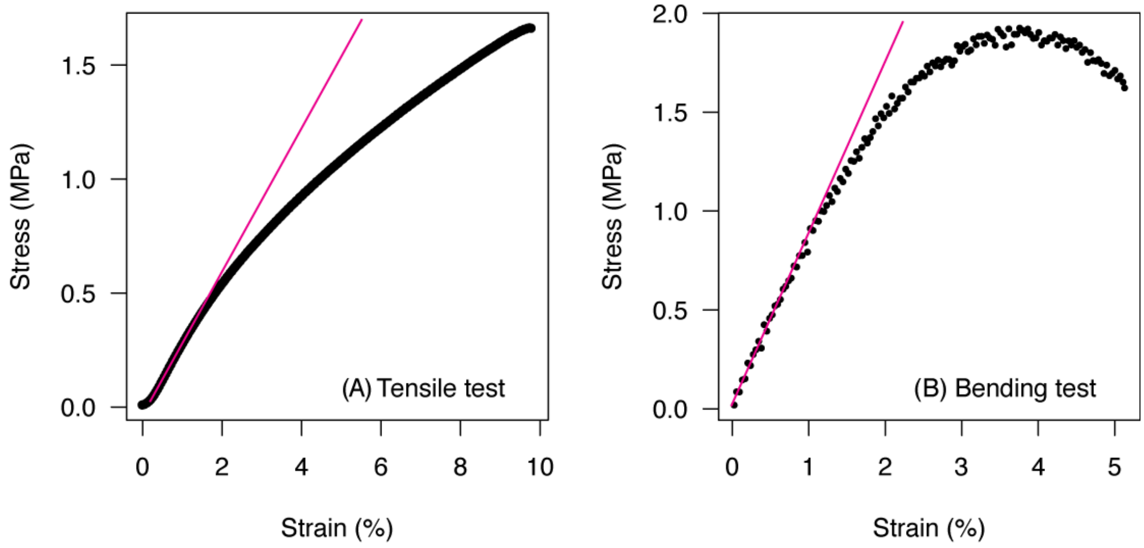

Fig. S2. A typical stress/strain curve for tensile tests (A) and bending tests (B) of leaf lamina (*Hedera helix*). The Young's modulus was calculated from the initial slope of the curve (red solid line).
